# First-in-human use of the dual-energy SMARTTOUCH™ SF radiofrequency and pulsed field ablation catheter for ventricular arrhythmia treatment

**DOI:** 10.1007/s10840-025-02219-0

**Published:** 2026-01-02

**Authors:** Shivaraj Patil, Konstantinos C. Siontis

**Affiliations:** Department of Cardiovascular Medicine, Mayo Clinic, MN Rochester, USA

**Keywords:** Premature ventricular contraction, Pulsed field ablation, Radiofrequency ablation

## Introduction

Radiofrequency (RF) catheter ablation is a well-established and effective first-line treatment for premature ventricular contractions (PVCs). However, for cases refractory to conventional approaches, advanced techniques may be needed, including the use of low-ionic irrigation, bipolar ablation, ethanol venous ablation, needle ablation, and surgical ablation, among others [1]. The potential applications of pulsed field (PF) ablation for challenging ventricular targets are starting to emerge. Preclinical evidence suggests that sequential, colocalized RF and PF ablation may confer an advantage in complex intramural substrates due to larger lesion formation [2]. We present the first human application of a dual-energy RF and PF focal ablation catheter for the ablation of symptomatic outflow tract PVCs in a patient who had previously undergone unsuccessful RF ablation.

## Case

A 68-year-old female with non-ischemic cardiomyopathy, persistent atrial fibrillation with prior catheter ablation, peripheral vascular disease, and high-burden PVCs with symptoms of palpitations, dyspnea, and lightheadedness was referred for redo catheter ablation. In the previous procedure at another institution, the PVC was targeted with RF ablation at the posterior septal right ventricular outflow tract (RVOT) and at the anterior septal left ventricular outflow tract (LVOT) unsuccessfully. Coronary venous mapping was not performed due to inability to advance catheters distally into the coronary venous system.

### Investigational dual-energy RF and PF ablation system

The patient consented to repeat catheter ablation with the THERMOCOOL SMARTTOUCH^®^ SF Dual Energy Catheter incorporating Pulsed Field Energy (Biosense Webster, Inc part of Johnson & Johnson MedTech, Irvine, CA) under compassionate use approval by FDA and the Mayo Clinic Institutional Review Board. This is a novel, investigational, dual-energy, contact force-sensing catheter that delivers irrigated RF as well as high-voltage, biphasic, monopolar, microsecond PF using a proprietary configuration via the TRUPULSE™ Generator (Biosense Webster, Inc). Each PF delivery consists of a train of 24 PF pulses without R-wave gating.

### Pre-procedure testing

**Fig. 1:**
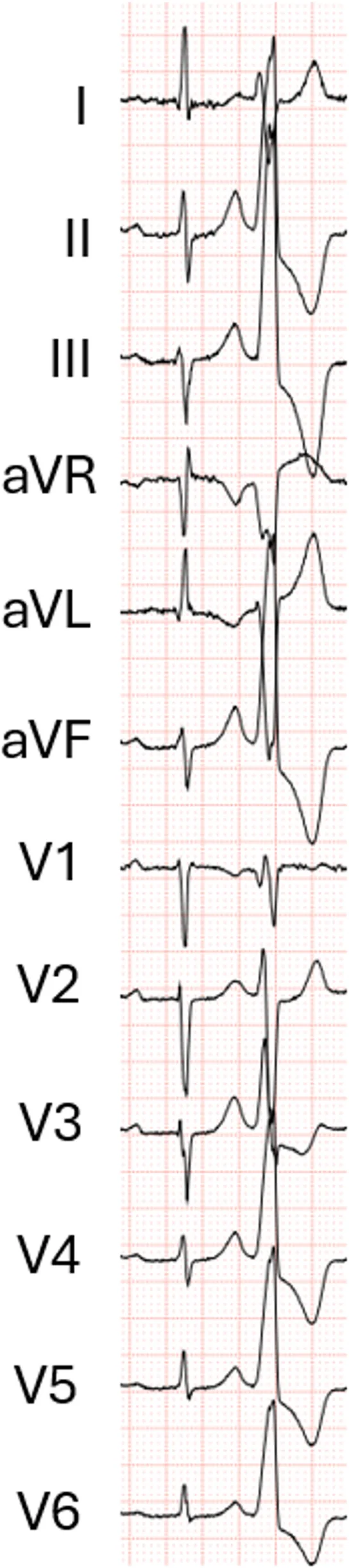
Target premature ventricular complex. A 24hr Holter monitoring demonstrated PVC burden 43% with a dominant PVC morphology of left bundle branch block, inferior axis and precordial transition at V3

Pre-procedure 24 h Holter monitoring demonstrated PVC burden 43% with a dominant PVC morphology of left bundle branch block, inferior axis and precordial transition at V3 (Fig. 1). Cardiac magnetic resonance imaging (CMR) demonstrated mid-myocardial late gadolinium enhancement (LGE) at the basal anterior and inferior septum, and the inferolateral left ventricle (LV). Left ventricular ejection fraction (LVEF) was quantified at 41%.

### Procedure description

The procedure was performed using monitored anesthesia care with deep sedation with propofol. Continuous heparin infusion was administered targeting activated clotting time > 300 s. Coronary venous mapping was unsuccessful due to inaccessibility beyond the proximal great cardiac vein despite multiple attempts and different catheters/wires utilized. Coronary sinus venogram demonstrated no contrast opacification beyond an apparent site of obstruction, without evident dissection.

**Fig. 2:**
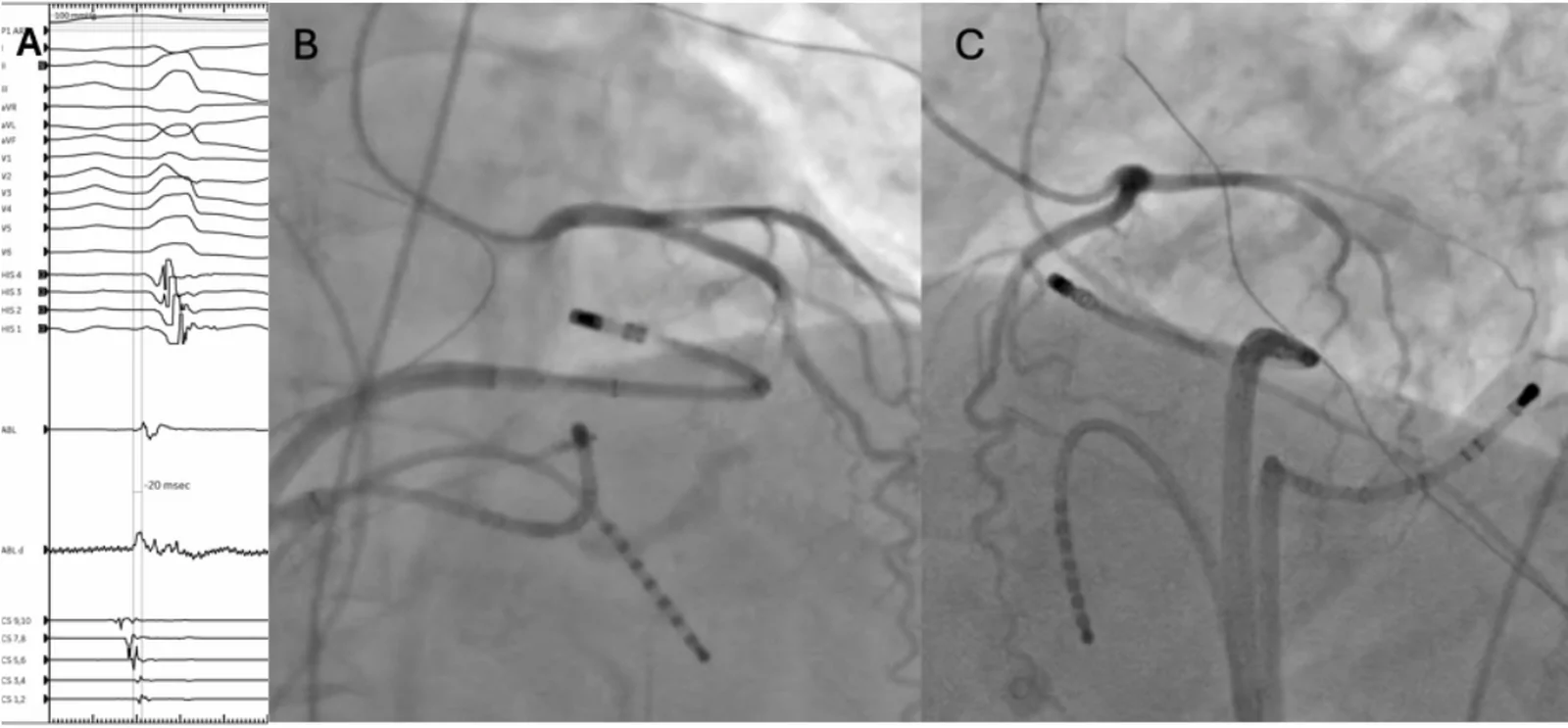
Intracardiac electrogram and coronary angiography with the ablation catheter at the earliest LVOT site. Sites of earliest activation with far-field appearing bipolar electrograms were noted immediately below the right aortic sinus near the commissure with the left aortic sinus (-20 milliseconds pre-QRS; Panel **A**), and at the juxtaposed septal RVOT (-11 milliseconds pre-QRS). A baseline intra-procedural left coronary arteriogram showed no significant abnormalities (Panels **B**-**C**, showing the ablation catheter at the earliest LVOT site)

**Fig. 3:**
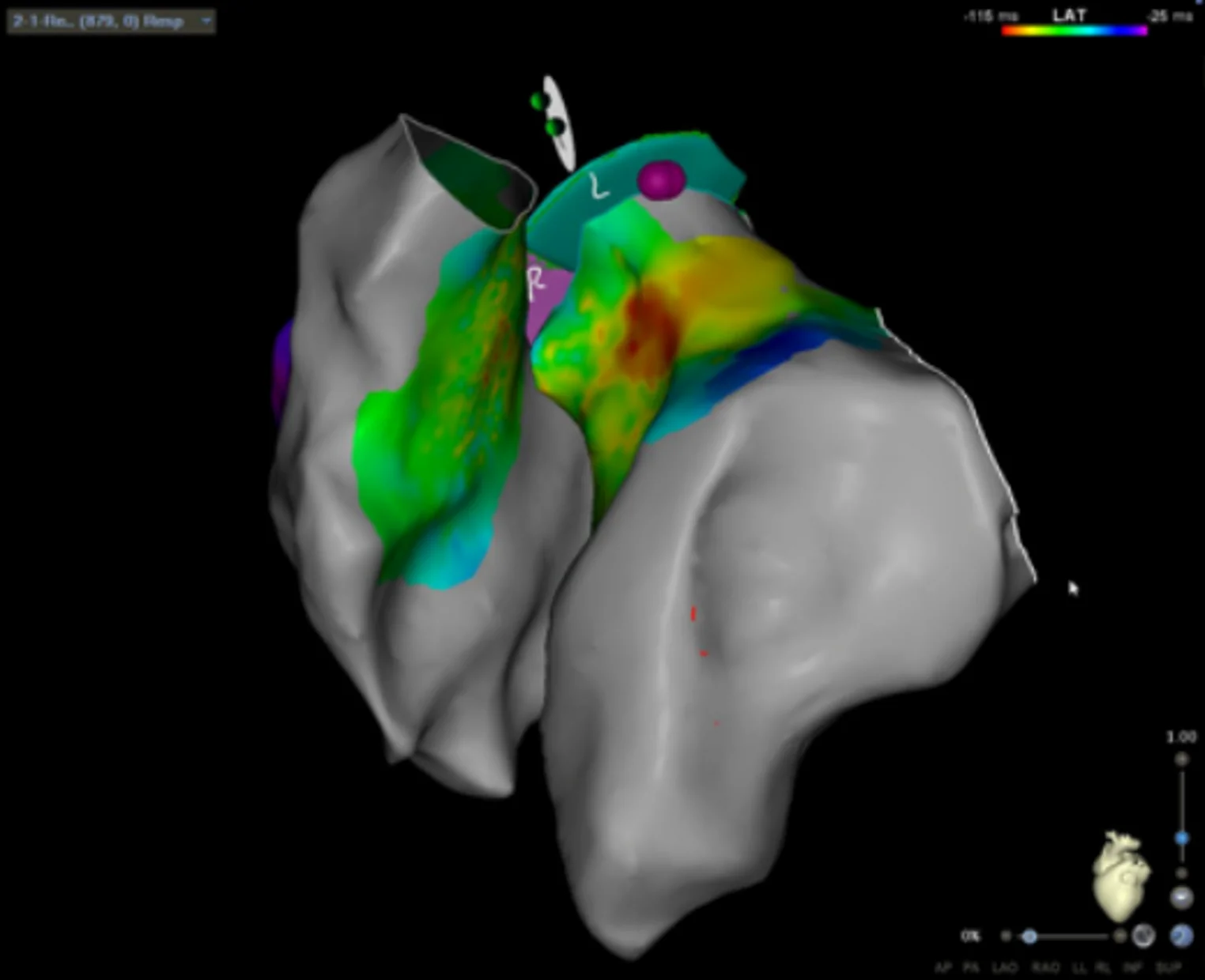
Electroanatomic activation mapping of the right and left ventricular outflow tracts. Activation mapping of the RVOT and LVOT was performed using a multielectrode catheter (OPTRELL, Biosense Webster, Inc). Overall, activation mapping was suggestive of intramural focus with broad breakout earliest in the LVOT

Substrate and activation electroanatomic mapping (CARTO 3) of the RVOT and LVOT was performed using a multielectrode catheter (OPTRELL, Biosense Webster, Inc). Additional point-by-point mapping was performed using the ablation catheter. Sites of earliest activation with far-field appearing bipolar electrograms were noted immediately below the right aortic sinus near the commissure with the left aortic sinus (-20 milliseconds pre-QRS), and at the juxtaposed septal RVOT (-11 milliseconds pre-QRS) (Fig. 2A). No early sites were identified in the aortic aspect of the right or left sinus. Overall, activation mapping was suggestive of intramural focus with broad breakout that was earlier in the LVOT (Fig. 3). Pace mapping at the RVOT and LVOT sites yielded at best modest correlation with the target PVC. An intra-procedural left coronary angiogram was performed for baseline assessment showing no significant coronary abnormalities (Fig. 2B-C).

Sequential, colocalized ablation was performed at the earliest LVOT site via transseptal access with RF (35–40 W for up to 1 min guided by impedance drop, electrogram diminution and lesion formation on intracardiac echocardiography) followed by PF (2–3 applications per site; 24 PF bursts per application [2, 3]) with minimum contact force of 5–10 g, resulting in only transient PVC suppression. Irrigation with normal saline was performed at a rate of 4 mL/min during PF delivery and 30 mL/min during RF delivery. No bubbles were noted with continuous intracardiac echocardiography monitoring during PF deliveries (Video 1). Systemic or intra-coronary nitroglycerin was not administered as pre-treatment. A coronary angiogram was performed after the first PF delivery and demonstrated no vasospasm. No apparent skeletal muscle recruitment or patient discomfort were observed during the PF applications. The LVOT ablation site was approximately 1 cm anterior to the annotated left-sided His - left bundle electrogram; no AV conduction abnormality was noted with PF delivery.

**Fig. 4:**
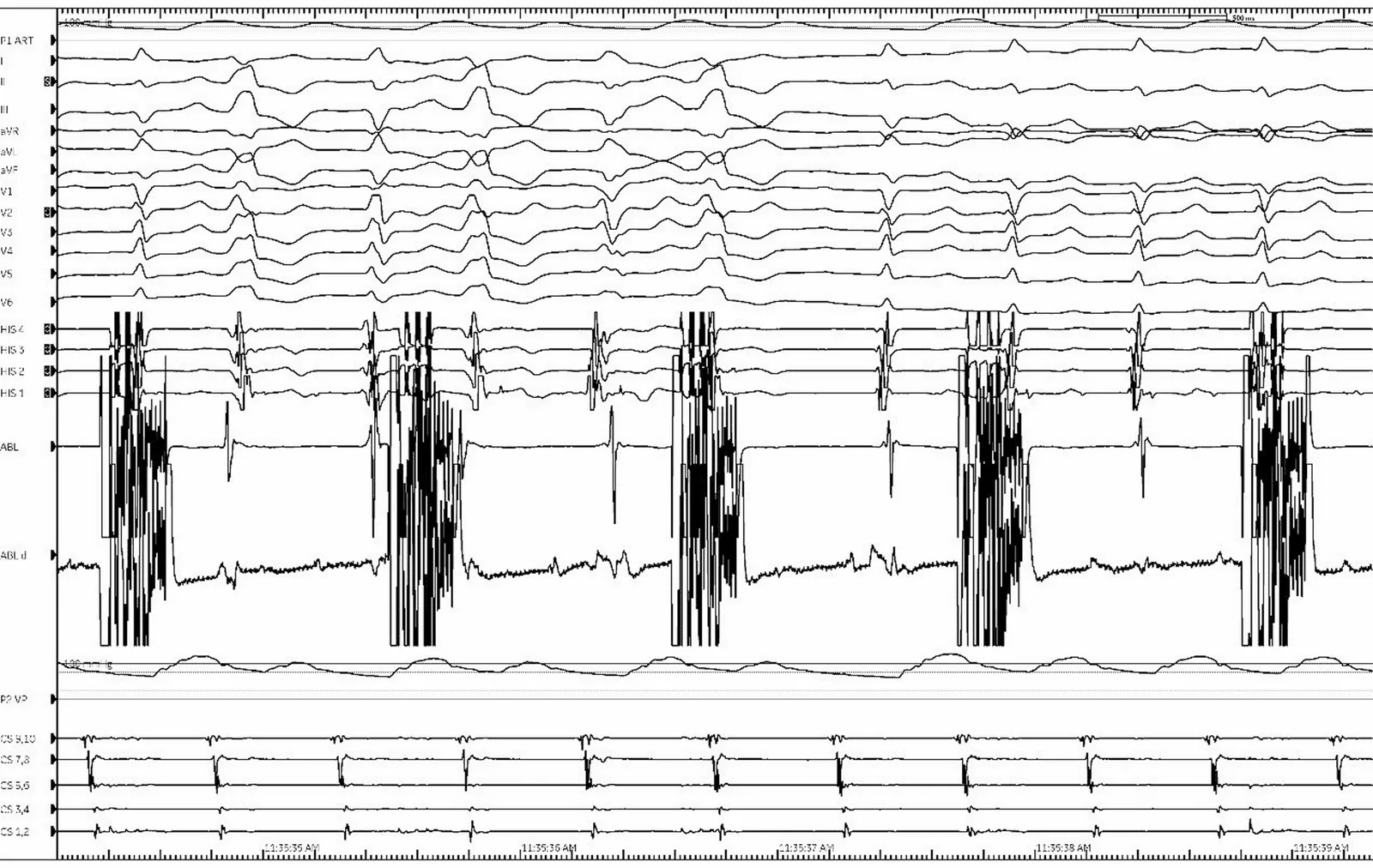
Suppression of PVC during PF application. A delivery of non-gated PF bursts at the earliest RVOT site is shown resulting in suppression of the PVC which, however, recurred and required further consolidative RF and PF ablation at the LVOT

**Fig. 5:**
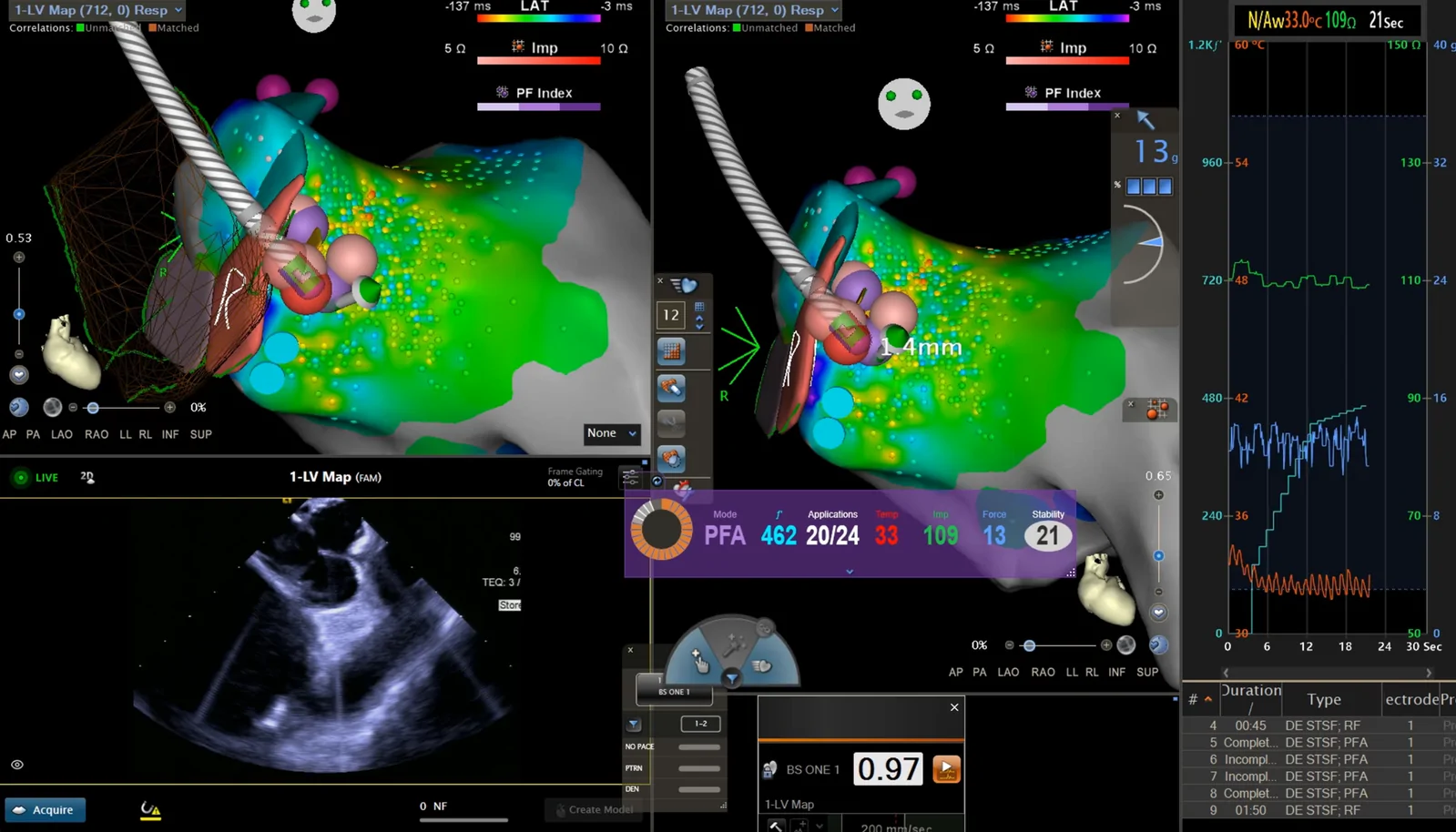
Carto3 interface demonstrating a PF delivery with the dual energy RF-PF ablation catheter at the septal LVOT. The ablation catheter was positioned via retrograde aortic approach at the septal LVOT where ablation with RF output of 40–45 W and longer duration up to 3 min, resulting in suppression of the PVC, followed by PF applications as shown in this snapshot and in Online Videos 2 & 3. The red/pink spheres represent RF lesions and the purple lesions represent PF lesions

**Fig. 6:**
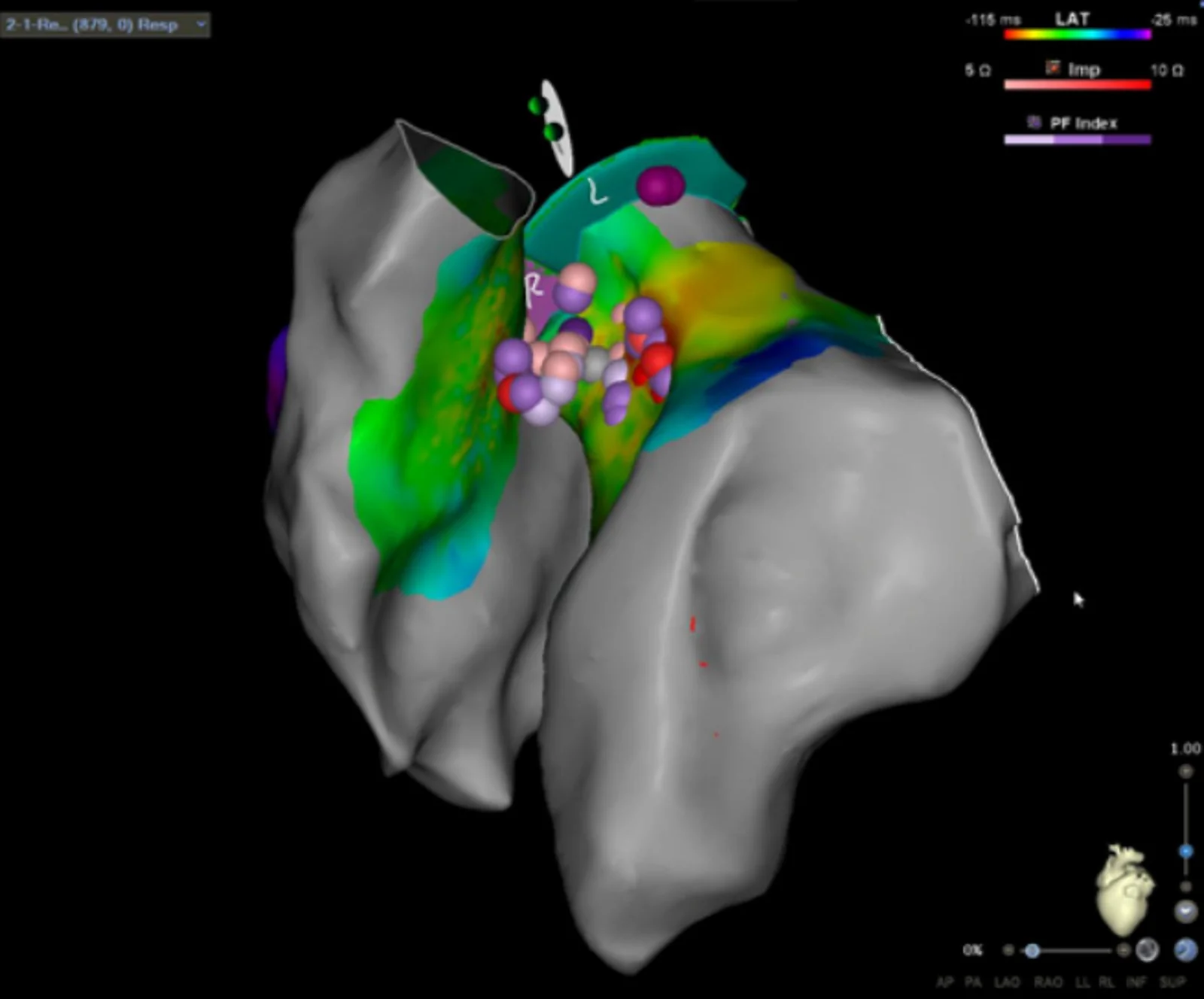
Final RF and PF ablation lesion set. In total, of 15 RF applications (*n* = 13 LVOT) and 23 PF applications (*n* = 19 LVOT) were delivered during the procedure. The purple spheres indicate PF lesions while the red/pink spheres represent RF lesions

Additional RF and PF ablation was performed in the septal RVOT targeting the earliest sites, albeit far-field, resulting in only transient PVC suppression with a slight change in morphology (Fig. 4). For improved catheter contact, the LVOT was then accessed via retrograde aortic approach and ablation was performed with higher RF output of 40–45 W and longer duration up to 3 min, resulting in suppression of the PVC, followed by PF applications (Fig. 5; Videos 2–3). Thereafter, no clinical PVCs were observed during a one-hour waiting period, with and without isoproterenol. In total, of 15 RF applications (*n* = 13 LVOT) and 23 PF applications (*n* = 19 LVOT) were delivered during the procedure (Fig. 6). The average PF index for all PF lesions was 447.4 ± 66.3.

### Post-ablation testing

**Fig. 7:**
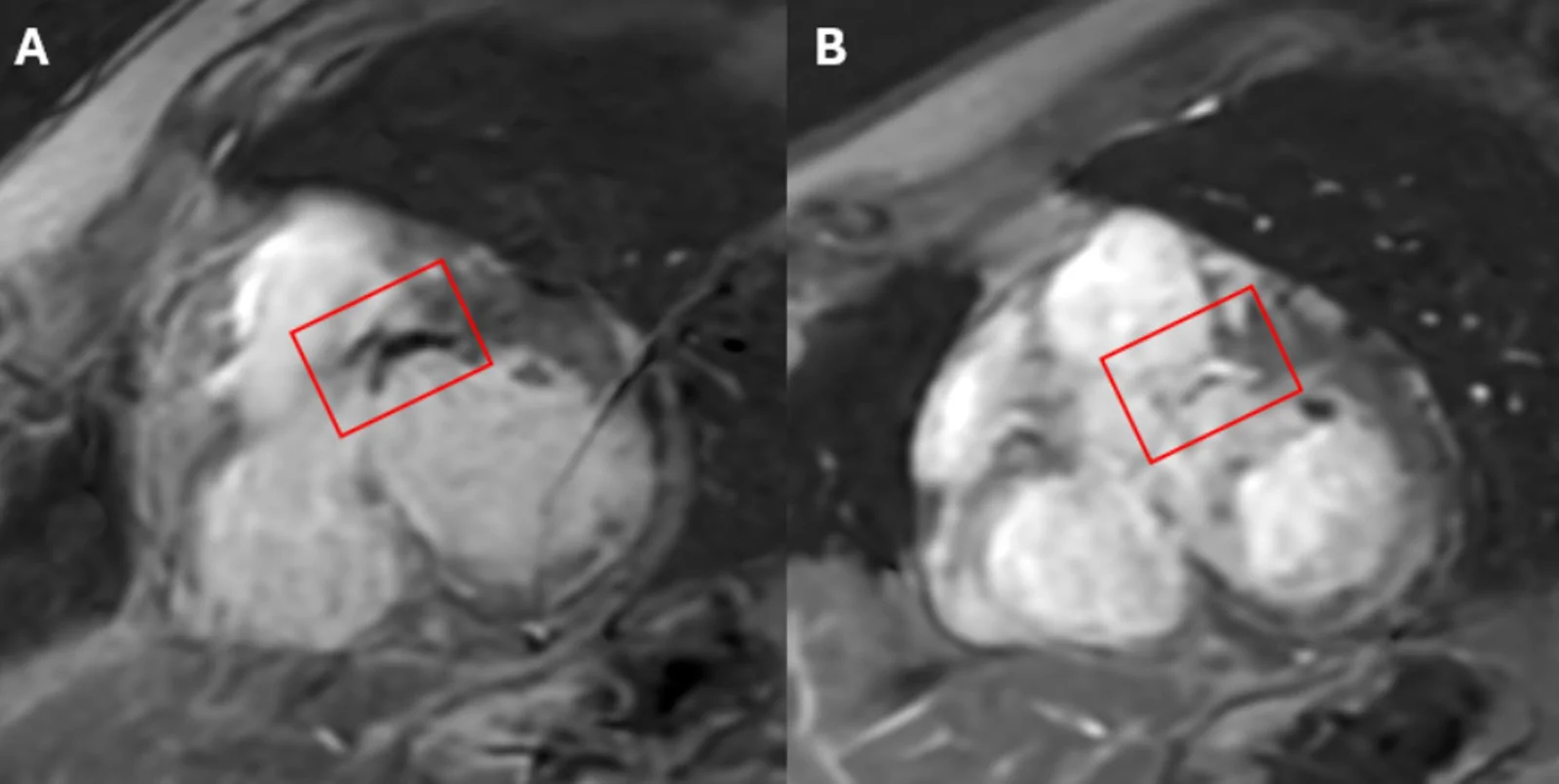
Post-ablation cardiac magnetic resonance imaging. CMR at 72 h showed confluent non-enhanced dark core of tissue on the septal outflow tract corresponding to the LVOT and RVOT ablation lesions (Panel **A**). At 2-month post-ablation follow-up, repeat CMR demonstrated near-transmural LGE in the basal outflow septum with significant contraction of the dark core region compared to the 72-hour CMR (Panel **B**)

No peri-procedural complications were recognized during a 24-hour observation post-ablation. There was no evidence of hemolysis with exception of a mild, non-specific lactate dehydrogenase elevation of 239 U/L (normal range 122–222 U/L). Peak troponin was 1207 ng/L 3 h after the procedure, declining to 663 ng/L the next day. CMR at 72 h showed confluent non-enhanced dark core of tissue on the septal outflow tract corresponding to the LVOT and RVOT ablation lesions (Fig. 7A).

At 2-month post-ablation follow-up, the patient reported resolution of her prior PVC-related symptoms. Repeat Holter monitor demonstrated a PVC burden < 1% and CMR demonstrated near-transmural LGE in the basal outflow septum with significant contraction of the dark core region compared to the 72-hour CMR (Fig. 7B). LVEF was 59% suggestive of prior PVC-mediated LV systolic dysfunction which improved after PVC suppression.

## Discussion

This first-in-human use of the investigational THERMOCOOL SMARTTOUCH^®^ SF Dual Energy Catheter incorporating Pulsed Field Energy for ventricular ablation highlights the feasibility of combining sequential focal RF with monopolar PF ablation as a potential tool for addressing challenging arrhythmias refractory to conventional RF ablation. In this case, targeting of intramural outflow tract PVC was accomplished with acute procedural and mid-term follow-up success, without any evident safety concerns. The safety profile observed in this case is consistent with emerging data on ventricular PF ablation [4]. There was no evidence of PF-induced coronary vasospasm, conduction system injury, ventricular arrhythmias, major hemolysis, or renal dysfunction in our case. Post-ablation CMR demonstrated near-transmural lesion formation acutely and at 2 months following ablation from both sides of the septum. Monitored anesthesia care was safely employed in our case, thereby avoiding the suppressive effects of general anesthesia on intraprocedural PVCs.

A challenge in ablating intramural ventricular arrhythmias is the creation of sufficiently large lesions that can eliminate a deep-seated target. In such cases, non-standard ablation strategies like low ionic solution irrigation, bipolar or simultaneous unipolar ablation, venous or intra-arterial ethanol ablation, wire ablation, intramural needle ablation, and non-invasive radioablation may be considered. These approaches have varying efficacy and safety profiles, while they also require specialized technical expertise, equipment and other resources. The optimal ablation strategy to treat ventricular arrhythmias with complex intramural substrates remains undefined, but the addition of focal PF to existing RF catheter platforms may represent an effective approach that can be easily integrated into current ablation workflows with which electrophysiologists are already familiar. In our case, we also employed high-power, long-duration (HPLD) RF lesions at the LVOT [5], each followed by PF deliveries. The PVC suppressed and did not recur after the delivery of a HPLD RF lesion, thus we cannot be certain whether acute success could have been accomplished with RF alone. Nonetheless, this patient had undergone a previous standard-of-care RF-only ablation procedure which was unsuccessful.

A fundamental advantage of using a system that combines RF and PF deliveries is the ability to create deeper lesions as compared to those achievable with either modality alone. Initial RF delivery can reduce local impedance and cause tissue edema, thereby potentiating electrical conductance and enabling the PF field to penetrate deeper tissue layers [6]. Histologically, this creates a hybrid lesion with a core of contraction band necrosis surrounded by a hemorrhagic and transitional zone of electroporation-mediated injury. In a preclinical model, sequential colocalized RF followed by PF delivery using the ablation system described in this report resulted in significantly larger and deeper lesions. Specifically, the mean lesion depth and width for PF alone (PF Target index of 300) were 3.5 ± 0.6 and 5.1 ± 1.8 mm, respectively. Lesion depths for combined RF (target index 400) and PF applications (target index 300) were significantly greater versus PF alone (6.2 ± 1.8 mm for RF followed by sequential PF application on the same location) [2]. The 72-hour post-ablation MRI in our patient demonstrated the expected nonenhancement (dark core) due to microvascular obstruction or coagulative necrosis within the lesion whereas interestingly we did not observe the typical rim of hyperenhancement that corresponds to hyperemia conventional RF-only lesions. The absence of this hyper-enhanced rim may reflect the different lesion properties of RF-only vs. RF + PF lesions. Yet, the spatial resolution of clinical-grade MRI is not sufficient to completely define these details thus these findings should be interpreted with caution. Importantly, the LVOT and RVOT lesions progressed to near-transmural late gadolinium enhancement approximately 2 months post-ablation demonstrating the chronic RF + PF lesion evolution to fibrotic scar in this timeframe consistent with previous observations with a different PFA system [7].

Use of PF in treatment of ventricular arrhythmias offers additional potential benefits. PF results in more effective lesion formation in scarred myocardium, with improved lesion homogeneity and depth compared with RF [8]. Repeated PF deliveries at the same location can further enhance lesion depth and volume, which is necessary when ablating thick myocardium [9]. The ability to titrate and confirm adequate contact force in a focal-tip ablation system not only improves RF delivery but also PF delivery. A significant positive correlation between contact force and lesion depth has been demonstrated in pre-clinical models [3, 10]. Coronary vasospasm is a well-known risk, and prophylactic systemic or intracoronary nitroglycerin administration may be considered if the distance to a major coronary vessel is less than approximately 10 mm. If systemic nitroglycerin is used, pre-treatment with phenylephrine can mitigate hypotension.

## Conclusion

This report describes the first use of the THERMOCOOL SMARTTOUCH^®^ SF Catheter Incorporating Pulsed Field Energy catheter in the human ventricle in a patient with refractory intramural outflow septal PVCs refractory to prior RF ablation. Elimination of PVCs was accomplished successfully with sequential colocalized RF and PF ablation without apparent safety concerns, and with near-transmural LGE on the outflow septum noted on CMR several weeks post-ablation. This case builds upon preclinical investigations of dual RF and PF ablation in the ventricle and provides the feasibility basis for further prospective investigation of this and other focal PFA ablation systems in patients with challenging substrates.

## Supplementary Information

Below is the link to the electronic supplementary material.

Supplementary Material 1

Supplementary Material 2

Supplementary Material 3

## Data Availability

No datasets were generated or analysed during the current study.
